# Supplementary material for: Rapid determination of seismic influence field based on mobile communication big data—A case study of the Luding Ms 6.8 earthquake in Sichuan, China
Source: PLoS One. 2024 May 10;19(5):e0298236. doi: 10.1371/journal.pone.0298236 (PMC11086885; doi:10.1371/journal.pone.0298236)
Supplement: Attachment — Submitted filename: Reply to Reviewer plos.docx [file pone.0298236.s002.docx]

Dear Editor

1. I have made modifications to the manuscript layout according to the style requirements and template of PLOS ONE;

2. Added Data Availability to explain the data sources, including descriptions of the data sources in Figures 1, 3, 4, 5, 7, 10, and 11;

3. Confirm the references to Figures 1 and 2 in the manuscript;

4. Upload the image file as a separate file;

5. Provided a minimum dataset;

6. Reply to the questions raised by Reviewer 1 and Reviewer 2

Thank you to Reviewer 1 for providing valuable suggestions for the article and providing explanations for Reviewer 1's suggestions.Improve the article based on the comments raised by Reviewer 2,I added a comparative plan in the "Conclusion" section of the article to reflect the advantages of the proposed plan, and found a professional translation agency to improve my English writing.

Kind regards,

Qingquan Tan

Dear Reviewer

Thank you very much for the suggestions from the reviewers.We have also done some work and exploration before. After the M7.0 Jiuzhaigou earthquake on August 8, 2017, we collected 10319900 mobile phone location records and 44600 spatial grid locations using big data. Based on the mobile phone data of 24 hr continuous positioning, this paper uses the mobile phone call location data to conduct quantitative analysis of the population in Jiuzhaigou Valley Scenic and Historic Interest Area disaster area from the time and geographical dimensions, estimates the deviation of multidimensional population distribution, and discusses the method of estimating the distribution of communication base station dropout in disaster area by eliminating the users of micro error data.

FIG .The destruction of communication big data of Jiuzhaigou M7.0 earthquake

Currently, the communication department has begun to pay attention to the impact of natural disasters and to build some high standard super base stations that can withstand common disasters. In addition, the disaster situation is most severe in the region, and it is possible that some areas have experienced the complete withdrawal of base stations. Due to the fact that mobile phones can still provide location information through satellite, WIFI, Bluetooth and other communication methods, there may still be a certain amount of communication location data within the disaster range in extreme cases.

There is a significant correlation between smartphone usage and population estimation bias. There are extreme situations in the earthquake stricken areas, with a small population in some northwest and southern mountainous areas, and a significant difference in personnel composition. The northwest mountainous areas are mainly composed of elderly people and children over 60 years old, with low smartphone usage rates and underestimated population values. The county seat near the epicenter is densely populated, mainly tourists, and the smartphone usage rate is high. If the unified model is used, the pre earthquake data will be overestimated to some extent. Different models were used for simulating the population of counties and mountainous areas in this study.

FIG .The destruction of communication big data during the Yangbi M6.4 earthquake

We have also done similar work on the 2021 Yunnan Yangbi 6.4 magnitude earthquake, accurately simulating the dominant direction of the earthquake impact field, reflecting the distribution position of macroscopic epicenters, and to some extent reflecting the attenuation trend of the earthquake impact field.

Rapid determination of earthquake impact field distribution based on mobile thermal data. By utilizing pre earthquake and post earthquake internet mobile phone positioning data in the earthquake area, the spatial distribution characteristics of disappeared mobile phone positioning data and the directional direction of their discrete point sets in the earthquake area can be analyzed, so as to timely and quickly reflect and determine the basic distribution of destructive earthquake disasters to a certain extent, and provide relatively accurate disaster distribution information for earthquake relief command.

Thank you to the reviewers for their professional suggestions.

Kind regards,
Qingquan Tan
